# Forgetting in chimpanzees (*Pan troglodytes*): What is the role of interference?

**DOI:** 10.1371/journal.pone.0234004

**Published:** 2020-05-29

**Authors:** Gema Martin-Ordas, Rebeca Atencia, Sofia Fernandez-Navarro

**Affiliations:** 1 University of Stirling, Stirling, United Kingdom; 2 Newcastle University, Tyne, United Kingdom; 3 Jane Goodall Institute, Arlington, Virginia, United States of America; Institut de Recerca i Estudis en Primatologia, SPAIN

## Abstract

Humans are constantly acquiring new information and skills. However, forgetting is also a common phenomenon in our lives. Understanding the lability of memories is critical to appreciate how they are formed as well as forgotten. Here we investigate the lability of chimpanzees’ short-term memories and assess what factors cause forgetting in our closest relatives. In two experiments, chimpanzees were presented with a *target task*, which involved remembering a reward location, followed by the presentation of an *interference task*—requiring the recollection of a different reward location. The *interference task* could take place soon after the presentation of the *target task* or soon before the retrieval of the food locations. The results show that chimpanzees’ memories for the location of a reward in a *target task* were compromised by the presentation of a different food location in an *interference task*. Critically, the temporal location of the interference task did not significantly affect chimpanzees’ performance. These pattern of results were found for both Experiment 1—when the retention interval between the encoding and retrieval of the *target task* was 60 seconds- and Experiment 2—when the retention interval between the encoding and retrieval of the *target task* was 30 seconds. We argue that the temporal proximity of the to-be-remembered information and the interference item during encoding is the factor driving chimpanzees’ performance in the present studies.

## 1. Introduction

Humans have a remarkable ability to store and recall information for long periods of time. However, we do not remember everything we encode; in fact, forgetting is a common phenomenon in our daily lives. Critically, it has been argued that ignoring or not being able to retrieve irrelevant or outdated information has an important adaptive value [1]. This is because forgetting avoids loading our memory storage with unnecessary information—which allows other memories to eventually be recorded and selected [1–3]. Forgetting—defined as “the inability to recall something now that could be recalled on an earlier occasion” [4, p.74]-is a common phenomenon in both short-term and long-term memory [5–7]. Thus, understanding what causes forgetting is critical if we are to understand how memory works [1].

In human research, two different mechanisms have been suggested to cause forgetting: *temporal decay* [8, 9] and *interference* [5]. Regarding the former, it is argued that memory traces decay over short amounts of time (e.g., 3 to 18 sec); that is, whereas one might be able to recall the to-be-remembered information after 3 sec, the amount of recalled information decreases as the delay increases. In this view, the absolute amount of time between encoding and retrieval is critical for forgetting to occur [10–12]. As for the second mechanism, the presence of an interference task can interrupt the consolidation process through which memory representations become more stable over time and more resistant to ongoing retroactive interference by similar material [13]. In particular, research has shown that an interference task (e.g., a list of words) induces more forgetting when it is introduced right after the to-be-remembered information compared to when the interference is presented later in the retention interval [5, 13, 14]. It is argued that the larger the temporal gap between the encoding of the to-be-remembered information and the interfering task, the more consolidation can occur [5, 15]. The question of which mechanism is at play in forgetting in short-term memory has been and still is object of debates [16]—with findings showing that time is what causes forgetting [17, 18] and others indicating that interference is the causal variable [19, 20].

Undoubtedly cognition requires preservation of and access to relevant information in memory. For example, remembering a series of digits when mentally calculating a sum would not be possible without short-term memory. Having a comprehensive picture of how short-term memory works involves understanding not only what factors drive forgetting in humans but also if the same factors are at play in other animal species—and, in particular, in our closest relatives. This piece of information is also critical to understand the evolution of the memory systems. Research on memory in great apes has mainly focused on the type of information that can be retrieved and for how long it can be remembered [e.g., 21, 22]. For example, great apes are able to remember information (i.e. tool locations) for intervals of up to 3 years [23]. Martin-Ordas and Call [24] also investigated the temporal course of great apes’ memory traces within 24 h after encoding and showed that, similar to humans, time and sleep play a critical role in the evolution of great apes’ memory traces. In other words, the authors found that there is a phase of several hours during which memories were vulnerable to interference, but poor memory retrieval and forgetting were ameliorated by the mere passage of time and sleep between encoding and retrieval. Fewer studies have focused on great apes’ short-term memories [e.g., 25–27] and not much research has investigated what mechanisms affect the temporal course of their memory traces within short retention intervals (e.g., 60 seconds). In humans, the duration of the short-term memory is surprisingly limited and forgetting has been reported to occur after very short retention intervals [e.g., 16].

The goal of the present experiments was to investigate chimpanzees’ memory processing in a short-term memory [28] reward-location paradigm that did not require training. Chimpanzees were presented with a *target task*, which involved remembering a reward location (e.g., left container), followed by the presentation of an *interference task*—requiring the recollection of a different reward location (e.g., right container). It was predicted that if the interference task disrupts the consolidation of the memory trace of the target task, chimpanzees will (1) make more mistakes in the target task when the interference task is presented right after the target task compared to when the interference task is presented later in the trial, and (2) misattribute the location of the reward in the target task by wrongly remembering the location associated with food in the interference task (retroactive interference) [5, 15, 29–32]

## 2. Experiment 1: Chimpanzees (i)

### (a) Material and methods

#### Subjects

Thirty semi-free-ranging chimpanzees (*Pan troglodytes*) housed at Tchimpounga Chimpanzee Sanctuary in the Republic of Congo (see Table 1) were tested. There were 14 females and 16 males with ages ranging from 4 to 30 years of age. Subjects were socially housed and most of them had access to large outdoor enclosures. Note that previous research has shown that these chimpanzees are psychologically healthy and they seldomly display stereotypical behaviours [33]. Subjects were tested individually. Subjects were not food-deprived, and water was available ad libitum through the testing times. See Supplementary information for details regarding housing conditions, feeding regimens, and environmental enrichment. The experiment received ethical approval from the Newcastle University of Medical Sciences Ethics Committee (Project name: Mechanisms underlying future-oriented behaviour) and strictly adhered to the legal requirements of the Republic of Congo, and had approval from the Ministere de l’Enseignement Superieur et de la Recherche Scientifique in the Republic of Congo.

**Table 1:** Name, gender, age (years) and study participation (Experiment 1 = 1; Experiment 2 = 2).

Name	Gender	Age	Experiments participated in
Elikia	M	30	1
Kola	M	20	1, 2
Talian	M	18	1, 2
Kimenga	M	15	1, 2
Castro	M	15	1
Tchimaka	M	19	1, 2
Limana	F	17	1
Lounama	F	17	1
Diba	F	23	1
Willi	M	4	1
Tchivindna	F	14	1, 2
Shilao	F	12	1
Luc	M	13	1
Tchibanga	F	20	1, 2
Ntsere	F	23	1
Koyamba	F	15	1
Makasi	M	9	1, 2
Likouala	M	11	1, 2
Tiki	M	17	1
Kaouka	M	11	1
Golfi	F	15	1
Fanituek	F	18	1
Leki	M	8	1, 2
Mvoti	F	16	1
Ngoro	M	13	1, 2
Poungou	M	10	1, 2
Manisa	F	11	1, 2
Yanco	M	10	1, 2
Isabel	F	29	1, 2
Ngouba	F	30	1
Chimpi	M	18	2
Petit Prince	M	7	2
Wolo	M	21	2
Petit Per	M	17	2
Tavich	M	17	2
Loufumbou	M	14	2
Timmy	M	19	2
Low	M	24	2
Kefan	M	18	2
Tambikisia	F	12	2
Motambo	M	9	2
Ouband	F	18	2
Mona	F	9	2
Moundele	M	9	2
Mbebo	M	8	2
Kudia	F	12	2

#### Apparatus and procedure

**Fig 1:**
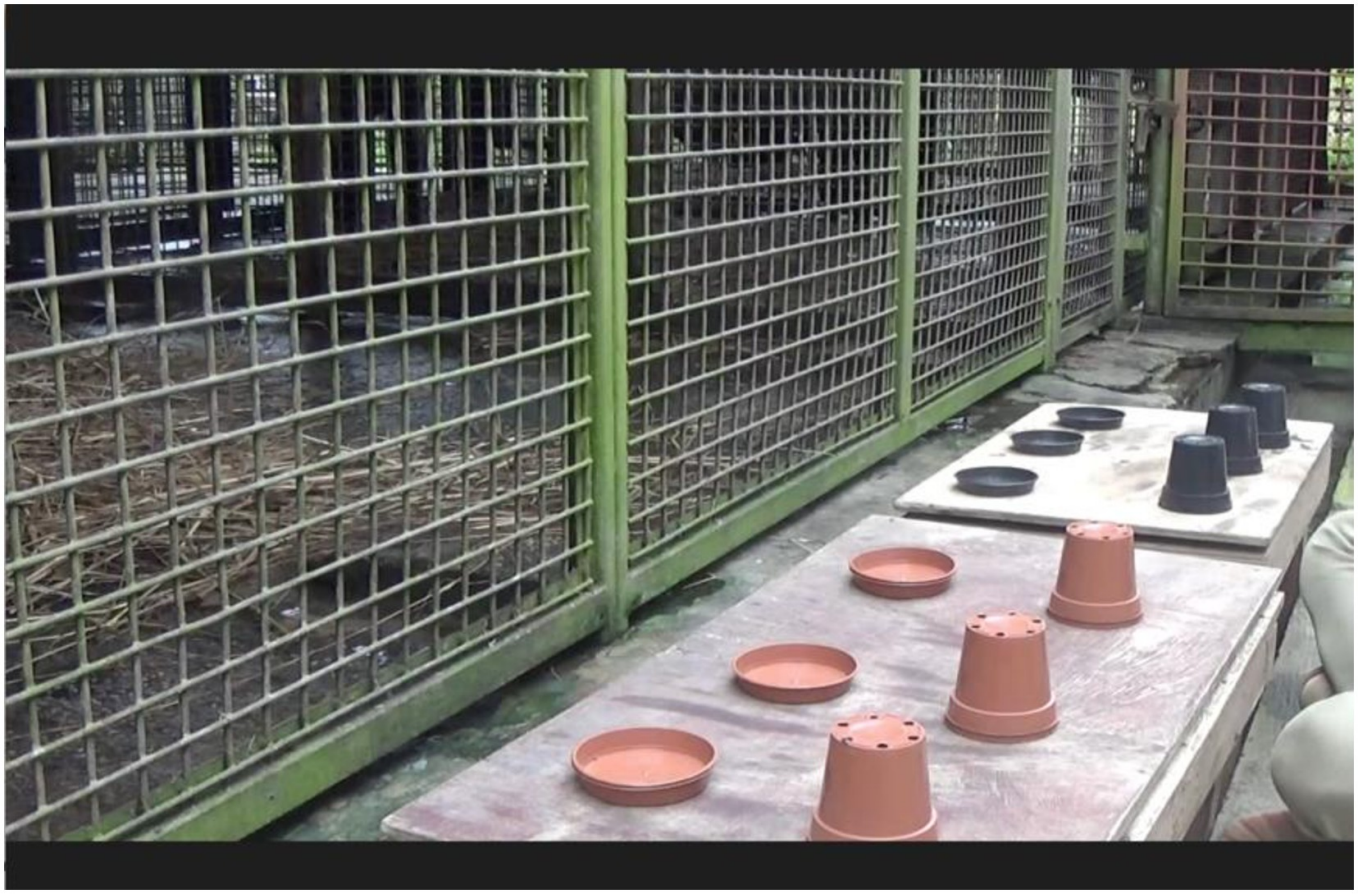
Setup for the experiment with the chimpanzees. The table located on the left side of the experimenter represents the target task and the table located on the right side of the experimenter represents the interference task.

The apparatus consisted of 6 opaque plastic containers—3 brown plant pots (10.5 cm diameter, 9.5 cm height) and 3 black plant pots (10.5 cm diameter, 9.5 cm height)—6 saucers—3 brown (internal diameter 11 cm, 2.5 height) and 3 black (internal diameter 11 cm, 2.5 height)—and two wooden platforms (70 cm x 35 cm). The experimenter (E) placed three containers on the platform about 30 cm apart centre-to-centre in front of a mesh partition each in front of a hole just above the platform (on E’s side; see Fig 1).

**Fig 2:**
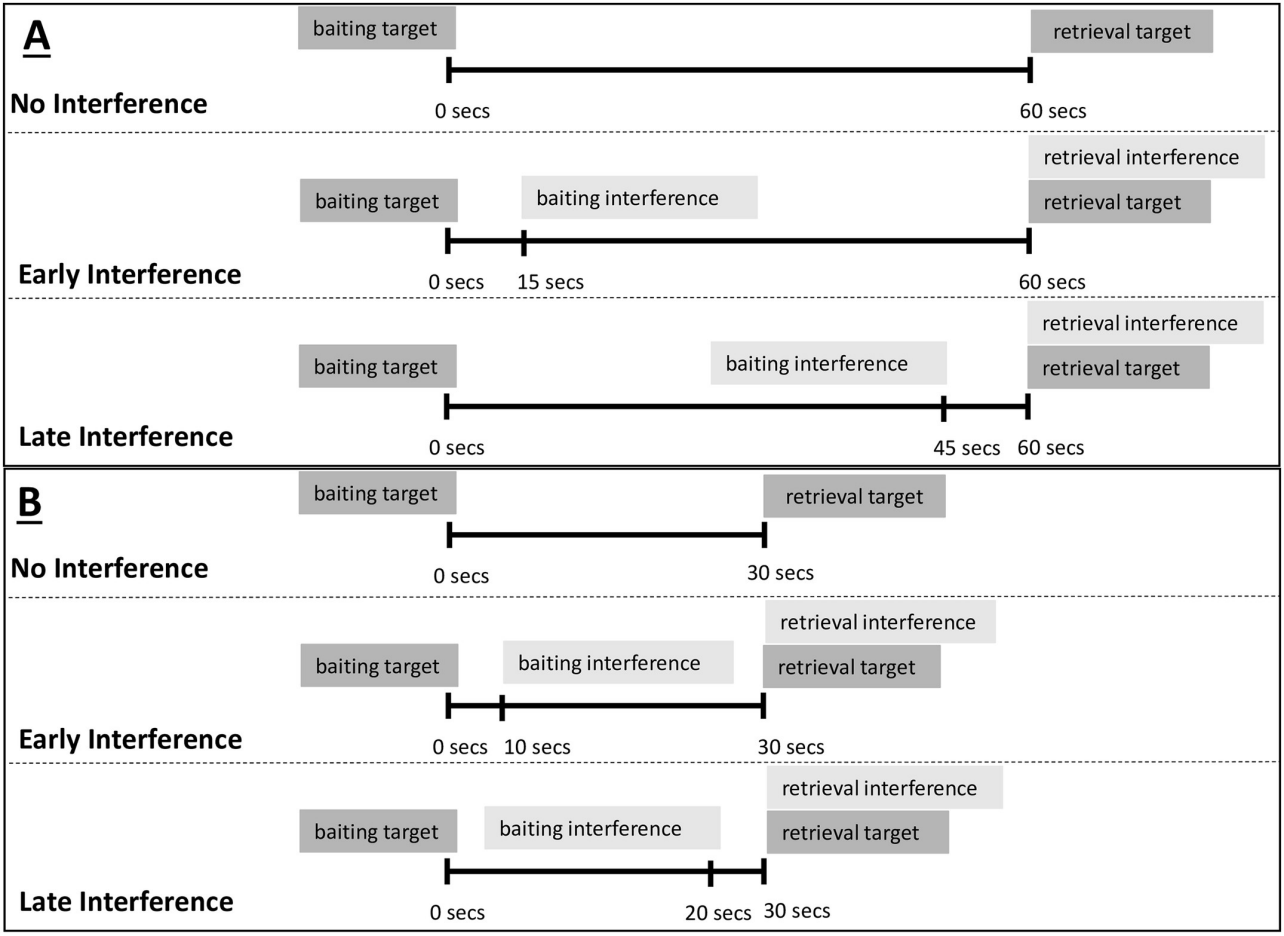
Schematic representation of each of the conditions and RIs for Experiment 1 (panel A) and Experiment 2 (panel B).

The E and the subject sat facing each other on either side of the mesh partition. The general procedure consisted of the E showing a reward (piece of banana) to the subject and placing it under one of the containers in full view of the subject. Following Martin-Ordas and Call [24], there were three conditions depending on the number of platforms baited and their timing (see Fig 2):

1. *No interference*. We presented subjects with *one* platform with three possible baiting places. Once E baited one of the containers with a reward, E waited 60 seconds before pushing the platform forward and letting subjects choose one of the containers. If subjects chose the correct container, E lifted up the container and gave subjects the piece of food. In contrast, if subject chose an incorrect container, first E lifted up the container and showed the subject the empty location; next she revealed the correct food location and removed the piece of food.
2. *Early interference*. In contrast to the *No Interference* condition, we presented subjects with *two* platforms (one for the target task and one for the interference task) located in the same testing room. First, E baited the target platform in the same way as in the *No Interference* condition described above. Fifteen seconds later, she baited the interference platform in the same way as she had baited the target platform except that the food was never placed in the same relative location in both platforms (e.g., never in the middle of the target platform and in the middle of the interference platform). Sixty seconds after the baiting of the target platform, E pushed the target platform and subjects chose one container. The E delivered the reward if subjects chose the correct container. If subjects chose one of the incorrect containers, E showed that the selected container was empty but did not show the correct location. The correct location was only shown at the end of the trial. Next, the interference platform was pushed forward and a choice was made. As before, E provided subjects with the reward if they chose the correct container. If subjects chose the incorrect container, E showed the subjects empty container as well as the correct location.
3. *Late interferenc*. We followed the same procedure as in the *Early Interference* condition with the only difference being that the baiting of the interference platform took place 15 seconds before the 60-seconds RI had elapsed (i.e., 45 seconds after baiting the target platform).

Subjects received a total of 6 trials in a single test session: 2 *No Interference*, 2 *Early Interference* and 2 *Late Interference*. There was a 15-sec inter-trial RI. All trials were done in one session and the number was kept to 6 in order to avoid learning or development of biases as well as to ensure that lack of motivation did not play a role in our tasks. The order in which subjects were presented with the three types of trials was randomized. The position of the food was counterbalanced across trials.

#### Analyses

Trials were recorded on video. Subject’s choice was counted as first container indicated by sticking the finger through the mesh (correct container = 1; incorrect container = 0). Data was coded for responses to target as well as for interference tasks. A second person coded 25% of the choices for inter-observer reliability. Inter-observer reliability was 100% for correct responses in both target and interference tasks. For the analyses, the percentage of trials in which subjects chose the container with the reward was calculated. We used non-parametric tests because the data was not normally distributed. To investigate the differences in performance among the *No Interference*, *Early Interference* and *Late Interference* conditions Friedman was used (Wilcoxon test was used as a post-hoc test). Wilcoxon test was calculated to determine chance levels (33.33% was the chance expected value), to investigate differences between *Early* and *Late Interference* conditions as well as to investigate subjects’ choices of containers. The types of errors that subjects made were also analysed. Wilcoxon test was used to determine whether subjects chose choose the container associated with food in the interference task (i.e., retroactive interference) and the container associated with food in the previous trial (i.e., proactive interference) at chance levels (33.33% was the chance expected value). Trial 1 and *No interference* condition were excluded from these analyses. This is because only retroactive interference could occur in Trial 1 and only proactive interference could occur in the *No Interference* condition. Note that also those trials in which both proactive and retroactive interference could be at play—because the location of food in the previous target task and in the interference task were the same- were not included in the analyses. All statistical tests were two-tailed.

### (b) Results and discussion

**Fig 3:**
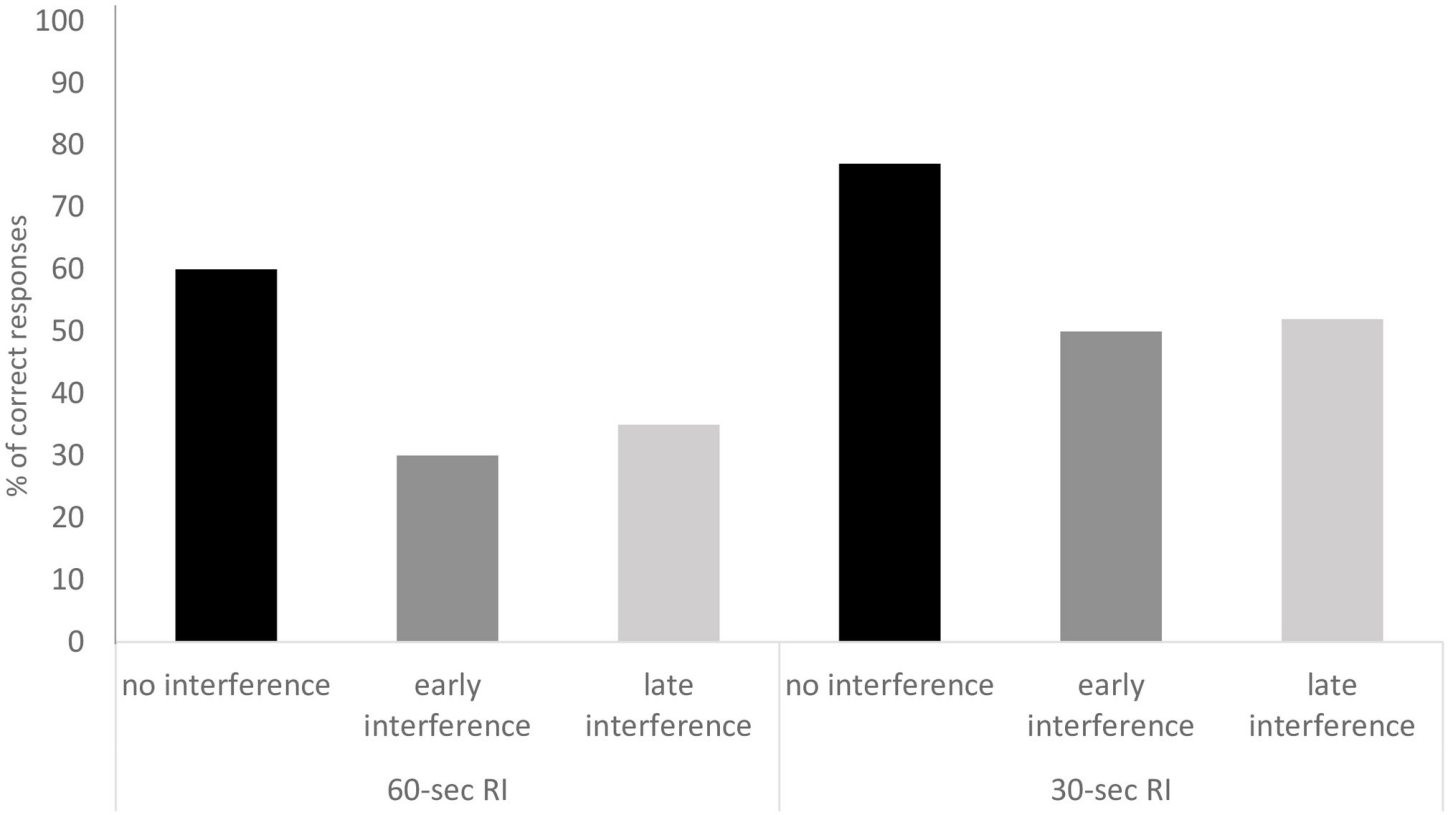
Percentages of correct responses for the target-task for the 60-seconds RI (Experiment 1) and 30-seconds RI (Experiment 2) trials as a function of the condition (No Interference, Early Interference and Late Interference).

Chimpanzees’ correct responses in the *target task* differed across conditions (χ^2^ = 13.23, df = 2, p = .001, n = 30; Fig 3): subjects better recalled the location of the food in the *No Interference* condition compared to the *Early* (*z* = 3.07, *p* = .002, *n* = 22) and *Late Interference* conditions (*z* = 2.98, *p* = .003, *n* = 16)—with subjects only performing significantly above chance in the *No Interference* condition (*z* = 3.65, *p* < .001, *n* = 30). Subjects did not perform better in the *Late Interference* compared to the *Early Interference* condition (*z* = .62 *p* = .544, *n* = 16).

**Fig 4:**
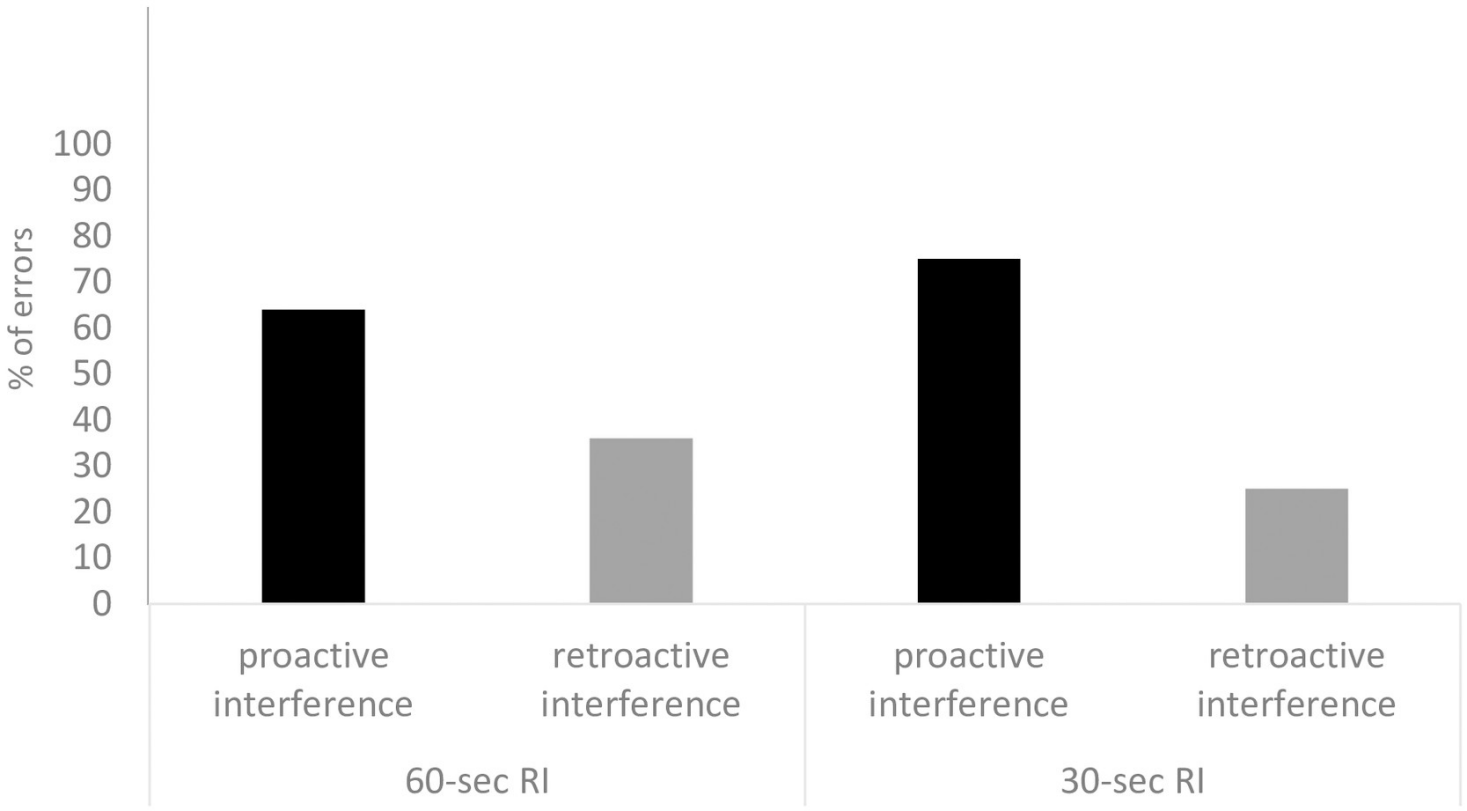
Percentages of errors caused by proactive interference (black bars) and retroactive interference (grey bars) for the 60-seconds RI (Experiment 1) and 30-seconds RI (Experiment 2).

Next, errors in the *target task* were analysed. Since no differences between *Late* and *Early Interference* conditions were found, subjects’ errors in both conditions were grouped. Of interest was if in the *target task* chimpanzees would choose more often the container associated with food in the interference task (i.e., retroactive interference) than the container associated with food in the previous trial (i.e., proactive interference). The results showed that when subjects made a mistake, they did not choose the container associated with food in the previous trial more often than the container associated with the food in the interference task (*z* = 1.41, *p* = .158, *n* = 20; Fig 4). However, subjects chose significantly above chance the container on the target platform associated with food in the previous trial (i.e., proactive errors; *z* = 2.87, *p* = .004, *n* = 24).

Experiment 1 showed that chimpanzees’ recall was impaired by the presence of an interference task. Strikingly, the temporal location of the interfering task—either early or later in the trial- did not significantly affect subjects’ performance. When mistakes were analysed, the information presented after the encoding of the target task (i.e., retroactive interference) did not influence chimpanzees’ performance more than the information presented before the encoding of the target task (i.e., proactive interference). Even though the present findings show that the presence of an interfering task affects the recollection of the target task, it was not found that the late interference task induced less forgetting than the early interference task. This is in contrast to what has been shown in the human literature [34].

**Fig 5:**
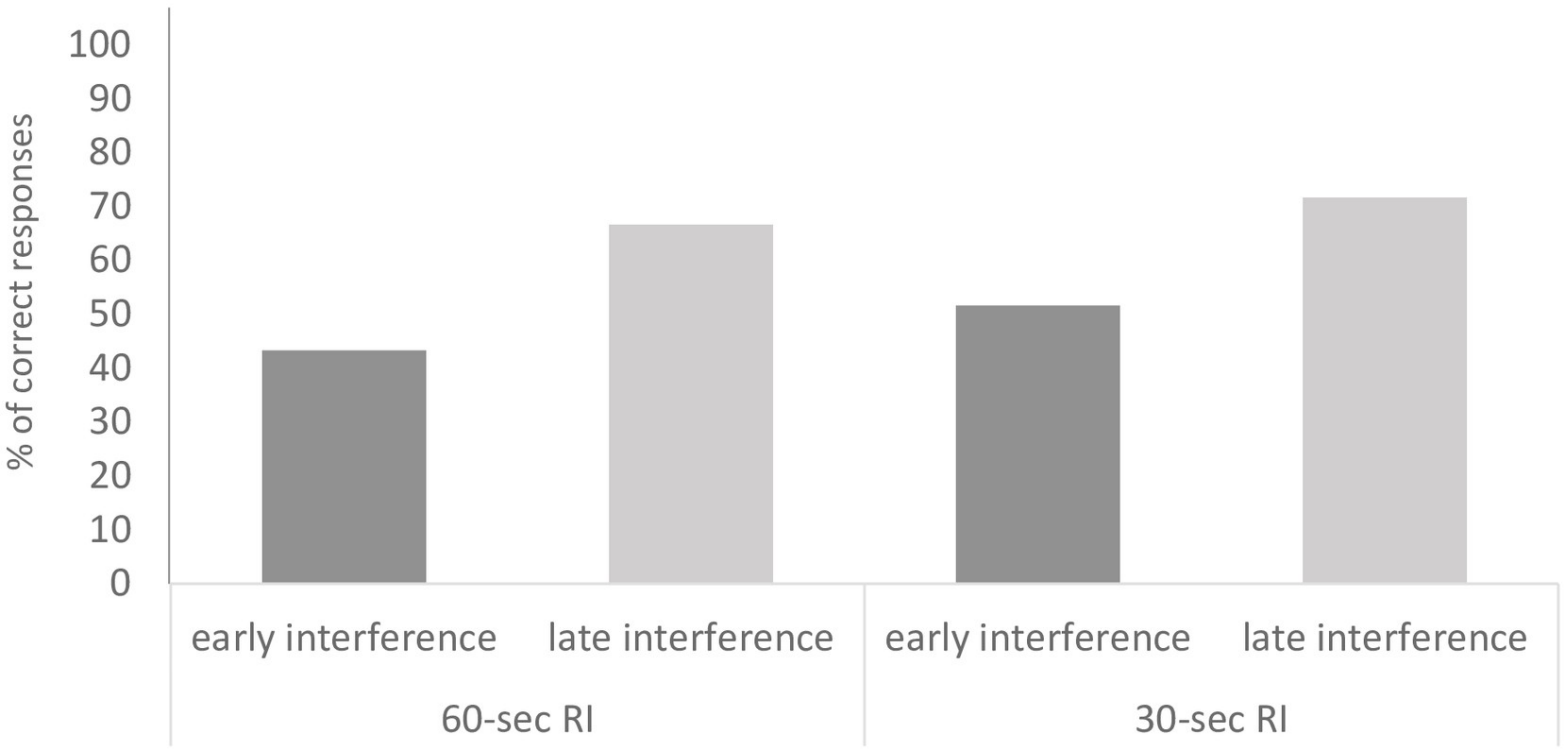
Percentages of correct responses for the interference-task for the 60-seconds RI (Experiment 1) and 30-seconds RI (Experiment 2) trials as a function of the condition (Early Interference and Late Interference).

It is true that subjects *only* performed significantly above chance in the *No Interference condition*, and as such, it is difficult to argue that chimpanzees’ memories decayed over time. In fact, when comparing the *No Interference* condition with the interference task in the *Early* (45 seconds RI) and *Late Interference* conditions (15 seconds RI)—which are the tasks that took place at different points during the RIs- significant differences across them were found (χ^2^ = 6.65, *p* = .036, *n* = 30). Chimpanzees tended to perform better in the *No Interference* (*z* = 1.87, *p* = .062, *n* = 18) and in the interference task of the *Late Interference* (*z* = 2.36, *p* = .018, *n* = 22) compared to the interference task of the *Early Interference* condition. Subjects were also significantly above chance in the interference task of the *Late Interference* condition (*z* = 3.88, *p* < .001, *n* = 30) but not in the interference task of the *Early Interference* condition (see Fig 5).

Altogether these findings suggest that chimpanzees were able to recall the information about the location of the food after 60 seconds—as performance in the *No Interference* condition indicates. Thus, these results cannot be explained in terms of memory decaying over this period of time. Still, memories might remain vulnerable to interference after 45 seconds and this could be why subjects’ recollections were still affected by the presence of an interfering task in the *Late Interference* condition. In humans, memory consolidation has been reported to occur a few seconds after encoding [e.g., 35, 36]. Experiment 2 investigated this issue by presenting chimpanzees with the same paradigm as in Experiment 1 but using shorter RIs (i.e., 30 seconds). Thus, using shorter RIs would allow us to investigate if the same interference effect as in humans is replicated. This is because the temporal distance between the target and interference tasks is shorter than for Experiment 1 and, therefore, might create more confusability between the to-be-remembered information [e.g., 37]. From a decay account, it would be expected that accuracy in memory recollection could benefit with shorter RIs [e.g., 16]. However, from the interference account, forgetting would happen regardless of the time passed between the presentation of the target and interference tasks.

## 3. Experiment 2: Chimpanzees (ii)

### (a) Material and methods

#### Subjects

Thirty semi-free-ranging chimpanzees (*Pan troglodytes*) housed at Tchimpounga Chimpanzee Sanctuary in the Republic of Congo (see Table 1) were tested. There were 8 females and 22 males with ages ranging from 7 to 24 years of age. As in Experiment 1, subjects were tested individually, were not food-deprived, and water was available ad libitum through the testing times. See Supplementary information for details regarding housing conditions, feeding regimens, and environmental enrichment. The experiment received ethical approval from the Newcastle University of Medical Sciences Ethics Committee (Project name: Mechanisms underlying future-oriented behaviour) and strictly adhered to the legal requirements of the Republic of Congo, and had approval from the Ministere de l’Enseignement Superieur et de la Recherche Scientifique in the Republic of Congo.

#### Apparatus and procedure

The same apparatus as in Experiment 1 was used for Experiment 2 (see Fig 1). The general procedure was the same as the one used in Experiment 1 with the main difference being that in Experiment 2 trials lasted 30 seconds from baiting of the target task. Thus, in the *No Interference* condition, subjects waited 30 seconds between the baiting of the containers and choosing one of them. In the *Early Interference* condition, the interference platform was presented 10 seconds after baiting the target platform. Subjects were allowed to choose 20 seconds after the baiting of the interference platform. Finally, in the *Late Interference* condition, the baiting of the interference platform took place 10 seconds before the 30-seconds RI had elapsed (see Fig 2). Subjects received a total of 6 trials: 2 *No Interference*, 2 *Early Interference* and 2 *Late Interference*. There was a 15-sec inter-trial RI. The order in which subjects were presented with the three types of trials was randomized. The position of the food was counterbalanced across trials.

#### Analyses

Trials were recorded and analyses were the same as in Experiment 1. A second person coded 25% of the choices for inter-observer reliability. Inter-observer reliability was 100% for correct responses in both target and interference tasks.

### (b) Results and discussion

Chimpanzees’ correct responses in the *target task* differed across conditions (χ^2^ = 8.34, df = 2, p = .015, n = 30; see Fig 3). In particular, subjects better recalled the location of the food in the *No Interference* condition compared to the *Early* (*z* = 2.74, *p* = .006, *n* = 22) and *Late Interference* conditions (*z* = 2.11, *p* = .034, *n* = 20)—with subjects’ performance tending to be significantly above chance in the 3 conditions (*No Interference*: *z* = 4.30, *p* < .001, *n* = 30; *Early Interference*: *z* = 2.50, *p* = .012, *n* = 30; *Late Interference*: *z* = 1.82, *p* = .068, *n* = 30). However, chimpanzees’ performance did not differ between *Late Interference* and *Early Interference* conditions (*z* = .19 *p* = .843, *n* = 19). Next, errors were analysed and, as in Experiment 1, errors in *Late* and *Early Interference* conditions were grouped. In this case, chimpanzees made more proactive than retroactive interference errors (*z* = 2.32, *p* = .020, *n* = 15; Fig 4).

Overall, Experiment 2 replicated the findings from Experiment 1—showing that chimpanzees’ recollection was affected by the presence of an interfering task- and extended it to shorter RIs (i.e., 30 sec). As in Experiment 1, subjects also performed significantly above chance in the *No Interference* condition—indicating that failing to recollect in the *Interference* conditions was not due to memory decay. However, the speed of memory consolidation does not seem to account for the current findings since the temporal location of the interfering task did not have a significant effect on subjects’ performance. When comparing the *No Interference* condition with the interference task in the *Early* and *Late Interference* conditions, significant differences were found (χ^2^ = 6.31, *p* = .042, *n* = 30)—with chimpanzees performing better in the *No Interference* (*z* = 2.64, *p* = .008, *n* = 19) and in the interference task of the *Late Interference* (*z* = 1.97, *p* = .048, *n* = 17) compared to the interference task of the *Early Interference* condition. Subjects were significantly above chance in the interference task of the *Early Interference* (*z* = 2.50, *p* = .026, *n* = 30) and *Late Interference* condition (*z* = 3.94, *p* < .001, *n* = 30; see Fig 5).

The results of Experiment 2 replicated the temporal pattern found in Experiment 1—with subjects performing better only after the longest (i.e., *No Interference condition*: Experiment 1, 60 seconds; Experiment 2, 30 seconds) and shortest (i.e., *Late Interference condition*: Experiment 1, 15 seconds; Experiment 2, 10 seconds) RIs.

## 4. General discussion

Chimpanzees remember the location of a reward after very short RIs, however recollection was hindered by the presence of *interference* tasks. Results also showed that when making mistakes, subjects tended to choose more often the container associated with food in the previous trial (i.e., proactive interference) rather than the container associated with food in the interference task (i.e., retroactive interference).

The findings presented here are in contrast with those found in the human literature of short-term memory consolidation [e.g., 8, 14, 35, 38–41]. As already mentioned, the temporal location of the interference task—either soon after the encoding of the target task or later in the trial- did not have a differential effect on subjects’ recollection for the location of the reward. These results were found regardless of the duration of the trial (i.e., 30 seconds or 60 seconds). Under the consolidation account, one would have expected that more forgetting occurred in the *Early Interference* condition compared to the *Late Interference* condition. This is because memories stay vulnerable right after encoding and tend to become resistant to interference with time. It is possible, though, that if we had presented the interference task immediately after the presentation of the target task, then the interference task could have had a stronger effect in chimpanzees’ recollections. Of interest is that in a delayed matching-to-sample study with pigeons, Calder and White [28] also failed to report evidence for consolidation in short-term memory when the delay between the task and interference was shorter than the ones used in the present studies (e.g., up to 12 seconds). Similar to the present findings, pigeons’ performance in Calder and White’s task was compromised by the presence of an interference task; however, their performance was not better when the interference happened at the end of the RI compared to when it happened at the beginning of the RI.

There are two possible accounts for the current findings. First of all, it is conceivable that it takes more than 45 seconds for chimpanzees to consolidate their memory traces—as such, the time window included in the Experiments 1 and 2 failed to capture the consolidation process. Interestingly, this would also indicate that the mechanisms driving consolidation might differ between human and non-human animals. This is because memory consolidation in humans have been reported to occur after very short RIs [e.g., 16]. However, the time required for a memory to be consolidated is still a matter of debate [e.g., 42]. It is plausible that some type of information (e.g., personal events) can be vulnerable to interference for long periods of time and that other type of information (e.g., visual information, motor skills) goes through much shorter consolidation periods—with some memory traces becoming stable more quickly than others [e.g., 13]. Accordingly, the information that chimpanzees were asked to remember in the present experiments (i.e., locations) might have been the type of information that remains labile for an extended period of time. Alternatively, it is equally possible that the effect of the interference task on the retrieval of information is related to an inhibitory process—as also reported in the human literature [e.g., 43–45]. Under this account, forgetting could have happened through an inhibitory process in which certain information (e.g., location of reward in the interference task) would have been more relevant than other (e.g., location of the reward in the target task) at retrieval—facilitating, then, the inhibition of the less important information [e.g., 43]. That is, forgetting could have resulted from a response to the interference instigated by the activation of competing information at retrieval. Future research could address this issue by presenting subjects with a highly desired reward in the target task and a less preferred reward in the interference task. One would expect that the different value of the rewards would facilitate the recollection of the correct location in both tasks.

The nature of the mistakes that subjects made when recollecting the location of the reward in the target task was also analysed. It is commonly argued that the acquisition/presentation of new information (i.e., interference task) during the consolidation process leads to the forgetting of the previously presented information (i.e., target task) [e.g., 5, 42]. Thus, when making mistakes, chimpanzees were expected to misattribute the location of the reward in the target task and wrongly remember the location associated with food in the interference task (i.e., retroactive interference). However, this is not what we found. Whereas in Experiment 1, no differences between retroactive and proactive interference were found, in Experiment 2 chimpanzees were more influenced by the location of the reward in the previous trial than in the interference task—we referred to this as “proactive interference.” Thus, rather than affecting the consolidation process—which is what retroactive interfere is argued to cause [46, 47]—in the present experiments interference might have affected chimpanzees’ act of retrieval [e.g., 48].

These results, then, could be explained in terms of *temporal distinctiveness* models—which have also been used to describe forgetting in human memory. These models predict that the amount of interference depends on the temporal proximity of the to-be-remembered information and the interference item during encoding [e.g., 49]: the smaller the temporal distance between the two items, the more they interfere with each other and the more they compete at retrieval [e.g., 49–52]. Consequently, temporally isolated items will be recalled better than temporally crowded items [e.g., 37, 49]. In the current experiments, it would have been expected that the information in the *Early Interference* conditions would be more difficult to recall than the information in the *Late Interference* conditions. This is because the target and interference tasks were more closely clustered. Critically, in the present experiments the levels of confusability in the target task could have been increased due to the short inter-trial interval (15 seconds). In fact, research with humans has shown that relatively long inter-trials intervals reduce the proactive interference as well as forgetting [e.g., 53–55]. Thus, the higher levels of proactive interference reported in the present studies could be due to the fact that information presented to the chimpanzees was not temporally distinct—causing lower performance in the target task as well as in the interference tasks of the *Early Interference* conditions.

In conclusion, the presence of an interference task compromised chimpanzees’ memories in a target task. Chimpanzees’ memories for the location of the food were affected by the interference task regardless of when this task was presented—either soon after the encoding of the target task or later in the trial. We argue that the time window in which the consolidation of this type of memories occurs might be longer than the RIs used in the current studies (e.g., 45 seconds). Similar to what it is found in humans, the nature of the interference errors found here support the temporal distinctiveness models. Future research should further investigate the extent to which temporal isolation and item crowdedness affect chimpanzees’ recollection in longer RIs. Similarly investigating whether interference tasks affect other type of memories (e.g., motor skills) the same way as reported here would be crucial to have a comprehensive picture of the consolidation process in our closest relatives. This information will be critical to understand what mechanisms drive chimpanzees’ forgetting and whether they are similar to those found in humans.

## Supporting information

S1 Data (XLSX)

S1 File (DOCX)
