# Supplementary material for: Forgetting in chimpanzees (Pan troglodytes): What is the role of interference?
Source: PLoS One. 2020 May 29;15(5):e0234004. doi: 10.1371/journal.pone.0234004 (PMC7259712; doi:10.1371/journal.pone.0234004)
Supplement: S1 File — (DOCX) [file pone.0234004.s002.docx]

**Tchimpounga Chimpanzee Sanctuary in the Republic of Congo**

**Background**

The Tchimpounga Chimpanzee Sanctuary is located in the Republic of Congo. The Sanctuary was established in 1992 by the Jane Goodall Institute. The aim is to provide lifetime shelter to orphaned chimpanzees mainly from West Africa.

**Animals**

Most of the chimpanzees were born in the wild and came to the sanctuary after being confiscated at early ages (~1-3 years old) due to the trade of chimpanzees as pets and bushmeat. Once the chimpanzees arrived at the sanctuary, they were raised by humans together with chimpanzees of similar ages. They were moved to a bigger social group when they were old and independent enough to do so. Tchimpounga Chimpanzee Sanctuary hosts approximately 120 chimpanzees.

**Enclosures**

All chimpanzees live in social groups. During the day the chimpanzees had access to large natural outdoor areas with trees and climbing structures. In the evening, all individuals come back from the outdoor enclosures and stay in dormitories (indoor enclosures) overnight. Subjects are tested in their indoor enclosures and their participation is voluntary.

**Diet**

Subjects were never food or water deprived. They were fed 3 times a day with fruits, vegetables and other suitable foods for chimpanzees.
